# Developing a Headspace SPME Arrow GC–MS Method for the Determination of Nicotine Biomarkers in Raw Sewage

**DOI:** 10.1002/jssc.70337

**Published:** 2025-12-18

**Authors:** Amir Salemi, Merve Çakmakci, Maryam Vosough, Torsten C. Schmidt

**Affiliations:** ^1^ Instrumental Analytical Chemistry and Centre for Water and Environmental Research Faculty of Chemistry University of Duisburg‐Essen Essen Germany; ^2^ Department of Chemistry Middle East Technical University, Çankaya Ankara Turkey; ^3^ IWW Water Centre Mülheim an der Ruhr Germany

**Keywords:** biomarkers, GC‐MS, green analytical chemistry, solid‐phase microextraction, wastewater

## Abstract

Wastewater‐based epidemiology (WBE) concerns the analysis of human biomarkers in untreated sewage samples as indicators of diseases, exposure to pollutants, and population lifestyle. Smoking, in various forms, and its prevalence in society are essential aspects of such studies. Anatabine, anabasine, cotinine, and hydroxycotinine are the most well‐known nicotine biomarkers, and the relation of their concentrations with the smoking prevalence has been used in WBE studies. Analysis of these compounds has been based on LC–MS following classical solid or liquid extractions. Regarding the complexity of the sewage samples, the final chromatograms are still plagued by a large number of contaminants, despite the use of tandem or high‐resolution MS detectors. In this work, a novel headspace SPME‐Arrow extraction method has been developed, optimized, and validated for determining four nicotine metabolites in raw sewage medium. A Box–Behnken design was used to assess the significance of temperature, pH and ionic strength as three factors influencing the extraction efficiency and also to optimize the experimental conditions. The effect of extraction time and its optimum value were determined individually. The optimized sample preparation method followed by GC–MS (SIM) was shown to be sensitive enough to analyze the analytes at their actual concentration levels (ng–µg L^−1^), thanks to ng L^−1^ LOQs and linear calibration up to 40 µg L^−1^. A raw sewage sample was also used to evaluate the accuracy and validate the method, with relative recoveries ranging from 87% to 103% for the spiked real sample at 400 ng L^−1^. The entire analytical procedure and the sample preparation step were assessed for their greenness, based on AGREE and AGREEprep models, respectively. Results showed that not only can this method be considered an alternative to SPE‐LC–MS methods, but it is also more environmentally friendly due to its automation and solventless nature.

## 1 Introduction

Untreated municipal wastewater is a highly complex matrix that includes various lifestyle‐ and exposure‐related biomarkers, whose occurrence, concentration, and spatiotemporal trends could provide essential clues for investigating human population activities, diseases, and the presence of chemical contaminants [1]. Excreted metabolites of caffeine [2], alcohol [3], illicit drugs [4], pharmaceuticals [5], pesticides [6], and nicotine [7] are a few examples of such biomarkers that are frequently used in wastewater‐based epidemiology (WBE).

Cotinine (COT), 3‐hydroxycotinine (HCOT), anatabine (ANT), and anabasine (ANB) are the main biomarkers of nicotine from the viewpoint of WBE studies [8, 9, 10, 11, 12, 13, 14, 15, 16]. COT and HCOT could be related to many forms of nicotine consumption, such as cigarettes, e‐cigarettes, and nicotine replacement therapy (NRT), while ANT and ANB are tobacco‐specific alkaloids and could only be related to tobacco smoking [8]. Regarding relatively high concentrations of COT and HCOT in wastewater, these compounds could be analyzed via direct injection into LC–MS/MS systems [14, 17, 18]. However, integrating solid‐phase extraction (SPE) into the analytical procedure could help improve method sensitivity and, at the same time, partially remove matrix constituents, specifically in raw municipal wastewater samples [10, 13, 19, 20]. On the other hand, ANB and ANT, which are considered minor tobacco consumption biomarkers with lower concentrations, are typically analyzed after sample preconcentration (using SPE) prior to chromatographic (LC–MS/MS) analysis [20, 21, 22].

The solid‐phase microextraction (SPME) Arrow represents a significant advancement in analytical chemistry, specifically designed for the automated determination of trace organic compounds in complex samples. It was developed to address key limitations of conventional SPME, primarily the fragility of its fibers and the comparatively small volume of the coated sorbent, which often restricted method sensitivity [23, 24, 25]. The SPME Arrow features a larger sorbent volume (e.g., 10.2 µL or 11.8  vs. 0.6 µL in conventional SPME, depending on the specific type), resulting in a substantial enhancement in sensitivity, often by an order of magnitude or more. Beyond increased sorbent capacity, a crucial advantage of the SPME Arrow is its superior mechanical robustness and stability. Its larger diameter (1.5 mm compared to approximately 0.7 mm for classical SPME) and arrow‐like tip facilitate easier penetration through the vial and GC septa, while also minimizing accidental bending and damage throughout the sample preparation procedure. The technique, just like the original SPME, also benefits from full automation, streamlining the entire sample preparation and introduction process, thereby reducing the risk of analyte loss and enhancing throughput and method greenness. Inheriting advantages from traditional SPME, it operates in a solvent‐free manner and allows for direct desorption into the chromatographic system. SPME Arrow has been successfully optimized for the determination of contaminants such as taste and odor compounds [26], phosphorus flame retardants [27], and fatty acids [28, 29] in water, volatile organic compounds in biological samples [30], and veterinary residues in food products [31], among other applications.

Several objective assessment approaches have been developed to evaluate the greenness of methods and support informed decision‐making. Green metrics quantify the environmental impact of analytical methods, ensuring sustainability and identifying opportunities for greening during method development [32]. In 2020, the Analytical GREEnness calculator was introduced [33] as a comprehensive evaluation tool, with assessment criteria derived from the 12 green analytical chemistry principles (SIGNIFICANCE). The overall performance score is generated by software, accessible at https://mostwiedzy.pl/AGREE. With the primary focus on the sample preparation step of the analytical procedure, AGREEprep [34] extends this concept by quantifying the environmental impact of sample preparation through 10 categories, recalculated to a 0–1 scale sub‐scores. Weighted input data generates an overall score ranging from 0 (*non‐green*) to 1 (*fully green*). The pictogram visually distinguishes individual criteria, allowing analysts to evaluate sample preparation from different perspectives and optimize experimental setups.

Several methods exist for quantifying nicotine biomarkers in wastewater, but most rely on classical SPE‐LC–MS or direct injection LC–MS/MS, which demand large sample volumes, extensive handling, and significant solvent use—yet still face matrix interferences from raw sewage. Moreover, few studies assess method greenness, automation, or measurement uncertainty. To address these gaps, this study introduces the first application of headspace SPME Arrow‐GC–MS for the simultaneous analysis of nicotine biomarkers in raw wastewater. The method was optimized using a Box–Behnken design (BBD) (temperature, pH, ionic strength, and extraction time), featuring fully automated, solvent‐free preparation, and was validated for uncertainty in accordance with the Eurachem/CITAC Guide (2012) [35]. Environmental impact was quantified using AGREE and AGREEprep. This approach provides a sensitive, robust, and environmentally friendly alternative to LC‐based methods, enabling accurate analysis in complex sewage samples while reducing environmental and operational impacts.

## 2 Materıals and Methods

### 2.1 Chemicals

The standard target analytes, as well as cotinine‐d3 (internal standard), were purchased from Sigma‐Aldrich (Steinheim, Germany). Pure water for sample preparation was produced by a PureLab Ultra water system (ELGA LabWater, Celle, Germany). Methanol (99.8%, Fisher Scientific, Loughborough, UK) was used to prepare stock solutions of 100 mg L^−1^ of each analyte, from which the other standard and spiked solutions were prepared. All standard and spiked samples were stored at 4°C prior to use. Sodium chloride (> 99.5%) was from Bernd Kraft (Duisburg, Germany) and used to adjust the ionic strength of the sample solutions.

### 2.2 Instrumentation

The target compounds were separated and quantified using a Shimadzu GC–MS‐QP2010 Ultra (Shimadzu Deutschland GmbH, Germany). The injector was equipped with a 2 mm i.d. × 5 mm o.d. × 95 mm length splitless liner from BGB Analytik (Böcken, Switzerland), and also a modified septum nut with a wider orifice was utilized to enable frictionless passage and penetration of the relatively thick SPME Arrow device. Thermal desorption was performed in splitless mode for 4 min at 250°C, followed by switching to split mode (1:10). An Rtx‐5 Amine column (30 m × 0.32 mm × 1 µm; Retek, Bad Homburg, Germany) was used for separation of the analytes, using helium (5.0, AirLiquide, Oberhausen, Germany) as carrier gas at a constant flow rate of 1 mL min^−1^. The temperature program started at 40°C (with a 3 min hold time) and was increased to 280°C at a rate of 30°C min^−1^, where it was maintained for an additional 15 min. MS transfer line and ion source temperatures were set to 280°C and 250°C, respectively. The following mass fragments were used in the selected ion monitoring (SIM) mode of the MS detector: **98**, 118, and 176 for COT; 79, 101, and **179** for COT‐d3 as internal standard; **106** and 192 for HCOT; **84**, 105, and 106 for ANB; and 54, 105, and **160** for ANT. The mass fragments shown in bold were used for quantification. For calibration (over the calibration range of 40–40 000 ng L^−1^) and validation (including spiked real sample analysis), the peak areas of the mass chromatograms were normalized against the peak area of the internal standard (mass chromatogram for 179).

### 2.3 Extraction Procedure and Optimization

The extraction process was performed using a PAL RTC autosampler (CTC Analytics AG, Zwingen, Switzerland). The SPME Arrow device (BGB Analytik, Böcken, Switzerland) was coated with a hydrophilic‐lipophilic balance/polydimethylsiloxane (HLB/PDMS) film, 120 µm thick, with a phase length of 20 mm. The extraction procedure began by transferring the 20‐mL septum‐capped sample vial containing 5 mL of the sample onto the heating/stirring plate. The sample (at pH values 7, 9, or 11) was conditioned for 10 min before extraction to reach thermal equilibrium and ensure complete dissolution of the added salt (0%–30% w/v, Table 1). The temperature was manually set according to the design of experiments (40°C–80°C), and magnetic stirring was maintained at a constant speed of 1000 rpm for all samples. After the conditioning period, the SPME Arrow penetrated the vial septum and extracted the analytes via headspace for 60 min. Then, the Arrow was withdrawn and thermally desorbed in the GC–MS inlet (4 min).

**TABLE 1:** Results of the analysis of variance (ANOVA) and the Box–Behnken design matrix.

ANOVA						
Source	Sum of squares	df	Mean square	*F* value	*p* value	
**Model**	0.6594	9	0.0733	52.71	< 0.0001	significant
*A* (temperature)	0.1026	1	0.1026	73.80	< 0.0001	
*B* (pH)	0.0904	1	0.0904	65.02	< 0.0001	
*C* (salt content)	0.0896	1	0.0896	64.46	< 0.0001	
*AB*	0.0007	1	0.0007	0.4819	0.5099	
*AC*	0.0002	1	0.0002	0.1390	0.7204	
*BC*	0.0250	1	0.0250	17.96	0.0038	
*A* ^2^	0.1750	1	0.1750	125.90	< 0.0001	
*B* ^2^	0.0721	1	0.0721	51.88	0.0002	
*C* ^2^	0.0692	1	0.0692	49.80	0.0002	
**Residual**	0.0097	7	0.0014			
Lack of fit	0.0066	3	0.0022	2.88	0.1667	Not significant
Pure error	0.0031	4	0.0008			
**Cor total**	0.6691	16				

Design matrix						
Factor		Level				
	−1	0	1			
Temperature	40	60	80			
pH	7	9	11			
Salt content (%)	0	15	30			

A BBD with 17 runs (including five center points) was selected to optimize the extraction procedure, with extraction temperature, pH, and salt content as the experimental variables. Design‐Expert (Stat‐Ease, Min, USA) was used to design the experiments and process the data. After performing the optimization experiments at the predetermined variable levels, the peak areas of the analytes were normalized using Microsoft Excel and then imported into the software along with the geometric mean of the normalized data. The normalization was performed by dividing the peak area of each individual analyte by its maximum value across the entire set of BBD experiments. The simultaneous extraction of the analytes was then optimized using the geometric mean as the main response to be maximized.

### 2.4 Real Sample

A raw municipal wastewater sample was collected from a treatment plant in Essen, Germany, and used to validate the optimized method. The sample was kept below 4°C during transport and filtered (25 mm, 0.22 µm pore size, polyvinylidene difluoride, BGB) upon arrival at the laboratory to be sterilized and refrigerated (at 4°C). Filter sterilization was performed to ensure operator safety and prevent microbial degradation of the samples. A part of the filtered wastewater sample was then spiked with the standard target analytes (400 ng L^−1^) and extracted to assess the relative recovery of the developed method.

### 2.5 Greenness Assessment

The AGREE was used as a tool to evaluate the greenness of the entire analytical procedure, and the AGREEprep was implemented to assess the sample preparation procedure specifically. Both software programs were downloaded from their respective websites [33, 34], and all the criteria were considered in their default weights. A recently published SPE‐Orbitrap LC–MS [16] method was compared with the developed HS‐SPME Arrow‐GC–MS method to evaluate the achieved greenness.

## 3 Results and Discussions

### 3.1 General Considerations

The highly complex matrix of raw municipal wastewater samples, along with the large number of different constituents, impacts the sensitivity and selectivity of the analytical method and necessitates extra cleanup steps. In case of SMPE, direct immersion of the extraction device into the sample leads to the sorption of these matrix interferents, which in turn, besides probable contamination of the device by non‐volatile compounds, could lead to a diminished sorption capacity of the sorbent as well as transfer of the undesired components into the GC system. One of the outstanding advantages of SPME over classical SPE is that it can be easily switched to the headspace mode and overcome the mentioned problems, provided that the analytes are volatile enough to migrate from the sample matrix into the headspace [36]. The other advantage of the headspace mode is that it allows the nearly independent variation of the sample composition by adding high concentrations of inorganic salts or adjusting the sample pH, without risking damage to the sorbing phase. In addition, it has been observed that the stirring rate of the sample could also be limited in direct immersion mode, while this rate has a significant influence on the kinetics of the extraction process [27]. Therefore, the headspace mode of extraction was selected throughout this study. The final point concerns the selection of SPME Arrow, which is commercially available with various sorbent coatings. Among these, hydrophilic‐lipophilic balanced (HLB) sorbents—one of the most recent developments in SPE materials—have demonstrated strong performance in extracting analytes across a broad range of polarities, as reported in the literature [37]. Based on these findings, the newly introduced HLB‐coated SPME Arrow was employed in this study. Consequently, the sorbent type was not treated as a variable for optimization.

### 3.2 Extraction Optimization

After completing the planned experiments, the peak area data for the compounds and their geometric mean values were normalized and imported into the optimization program. This step enabled the determination of the optimal factor levels for the highest geometric mean of the normalized responses, thereby optimizing the extraction performance for all analytes simultaneously. Table 1 summarizes the results of the ANOVA for the geometric mean of the responses, which reveal the significance of the model and the insignificance of the lack of fit (with *p*‐values of 0.0001 and 0.1667, respectively). Furthermore, the main factors were all significant in both first‐ and second‐order terms (*p* values < 0.05). At the same time, among the interactions, only the interaction between pH and salt concentration (BC) was significant.

**FIGURE 1:**
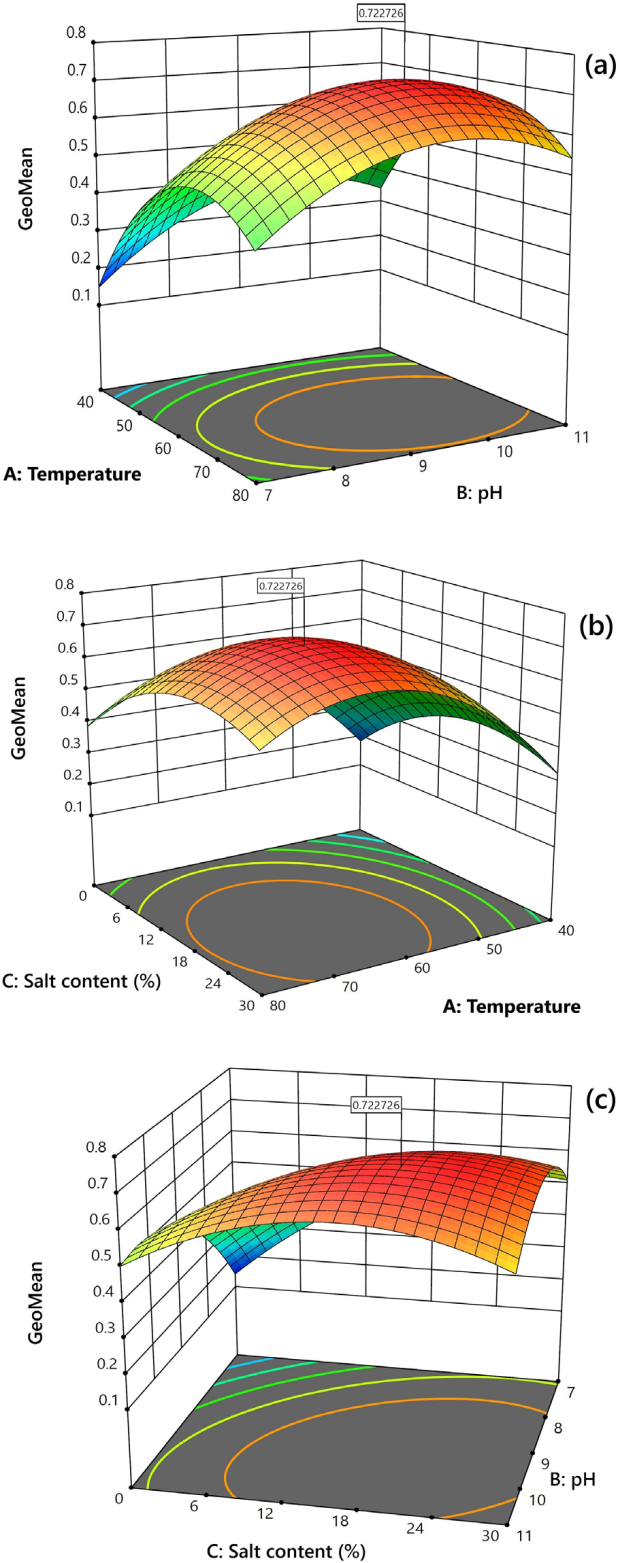
Response surfaces obtained from BBD with the geometrical mean of the normalized analyte peak areas (GeoMean) as the main response and (a) temperature–pH, (b) temperature–salt content, and (c) salt content–pH, as the variable pairs in each response surface.

The response surfaces in Figure 1 represent the variation in overall extraction performance for the target analytes (represented by the geometric mean) in response to the concentration of the dissolved salt (% w/v), sample pH, and extraction temperature. The second‐order effects of all variables, as well as the interaction between sample pH and salt content, are evident in these response surfaces. This interaction effect is observed in Figure 1c, as the different curvature of the opposite edges of the response surface. This interaction could arise from the fact that adjusting the pH of the sample alters the ionic strength of the solution due to the concentration of the added base. As a result, the sodium chloride concentration is not the only variable that affects the ionic strength, and the observed effect of the salt concentration varies with changes in the pH value.

**FIGURE 2:**
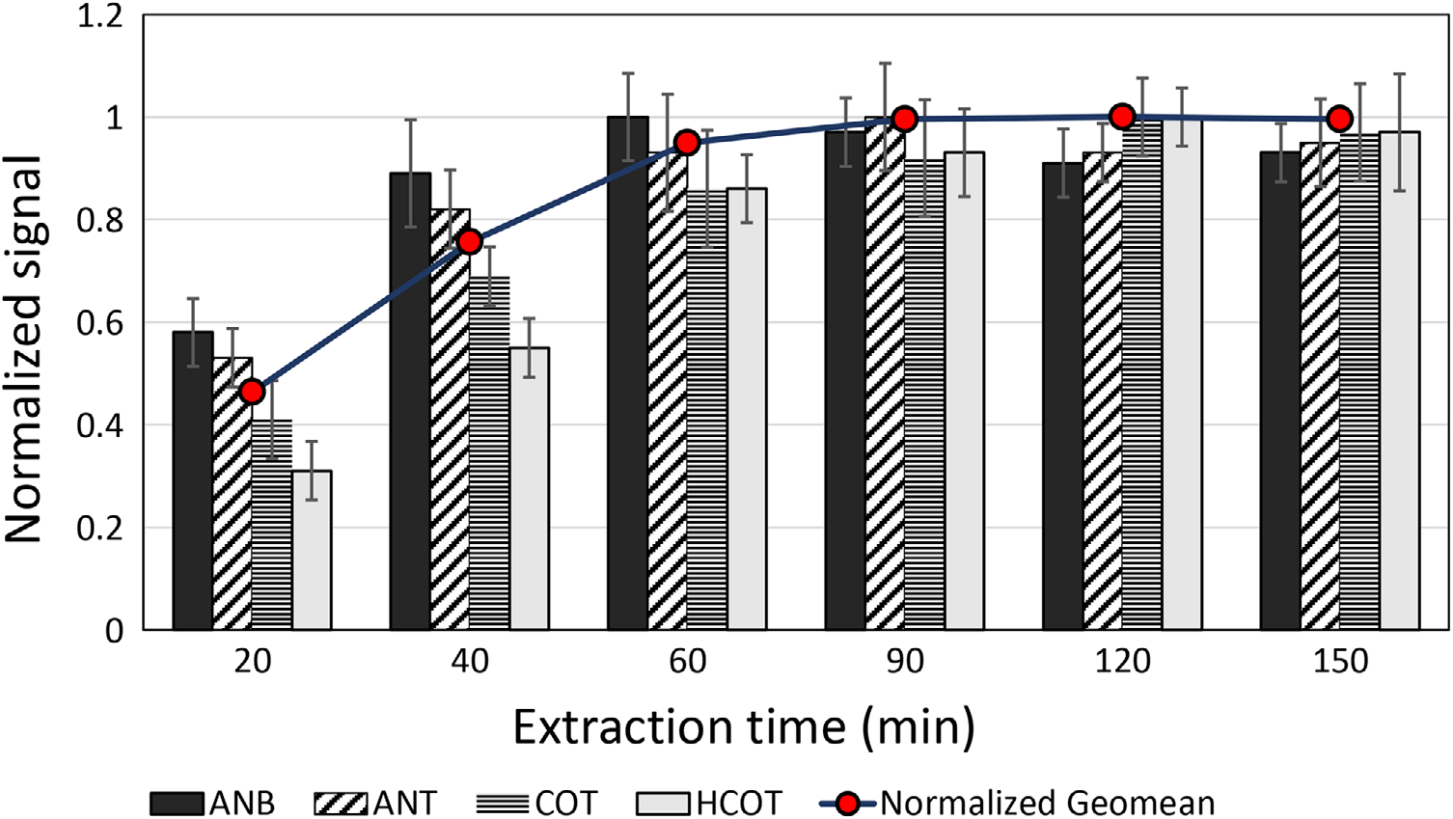
Effect of time on the extraction of nicotine biomarkers. The geometric mean of the normalized analyte peak areas is shown as circles overlaid on the bars.

The effect of extraction time was studied individually to observe the trend of extraction efficiency over a wider time range, as well as to identify the possible equilibrium or maximum point for each analyte. A set of triplicate extractions was performed at six different time points (20, 40, 60, 90, 120, and 150 min), as shown in Figure 2. The slope of the increase in extraction efficiency with time varied for the target analytes due to their volatility. Consequently, ANB reached a maximum at 60 min, followed by ANT (90 min), while COT and HCOT needed at least 120 min to reach the equilibrium. It was also observed that ANB and ANT, as the more volatile analytes, tend to decrease from their maximum values at higher extraction times. This phenomenon can be attributed to the competition between analytes with different affinities for the sorbent, leading to the displacement of those with weaker interactions [38]. The geometric mean of the peak areas of the target analytes was used again to find the optimum extraction time, as shown by the line drawn over the bars in Figure 2. Obviously, 90 min of extraction could provide the maximum efficiency, however, 60 min was selected in our study, to increase the speed of the overall analysis.

The above discussions confirm that the optimum extraction conditions could hardly be achieved using the one‐factor‐at‐a‐time method, using a reasonable number of experiments. The calculated optimum solution for the simultaneous extraction of COT, HCOT, ANB, and ANT was selected as follows: pH 10, 20% salt concentration, 65°C extraction temperature, and 60 min extraction time.

### 3.3 Analytical Performance Characteristics

A series of calibration samples at seven different concentration levels (40, 100, 400, 800, 1600, 8000, and 40 000 ng L^−1^ in ultrapure water with pH and ionic strength adjusted to the optimum levels) was prepared and analyzed in triplicate (Table 2) to calculate the calibration curves for each analyte. The resulting *R* ^2^ values demonstrated suitable linearity across the calibration range, spanning three orders of magnitude. In addition, the method's repeatability (intraday) was assessed using five replicate measurements of water samples (similar to the calibration set) at concentrations of 50 and 1000 ng L^−1^. As shown in Table 2, the relative standard deviation (RSD%) values ranged from 6.7% (ANB) to 10.3% (COT) at the lower concentration level and from 5.7% (ANB) to 7.7% (HCOT) at the higher level. Interday precision has also been calculated using replicate analyses of the pure water sample spiked at 400 ng L^−1^ over five consecutive days and the results ranged between 6.9% and 8.1%. Limits of detection and limits of quantification (LOD and LOQ) were calculated based on a signal‐to‐noise ratio of 3 and 10, respectively, at the lowest calibration concentration (40 ng L^−1^) point. The lowest LOD and LOQ were observed for ANB, and the highest values were observed for HCOT.

**TABLE 2:** Analytical performance characteristics of the optimized HS‐SPME Arrow GC–MS method.

	*R* ^2^ ^a^	LOD^b^	LOQ^c^	Precision intraday,^d^ %		Precision interday,^e^ %	Relative recovery,^f^ %	Precision of recovery,^g^ %	Uncertainty,^h^ %
		(ng L^−1^)	(ng L^−1^)	50 ng L^−1^	1000 ng L^−1^				
COT	0.9921	7.9	20.1	10.3	6.8	6.9	91	8.7	24.7
HCOT	0.9950	12.8	42.2	9.5	7.7	7.4	87	9.5	31.9
ANB	0.9914	3.2	10.6	6.7	5.7	8.1	103	7.7	19.4
ANT	0.9937	4.6	15.2	8.3	7.1	6.6	89	6.3	26.7

^a^ Over the calibration range of 40–40 000 ng L^−1^.

^b^ Based on *S/N* = 3.

^c^ Based on *S/N* = 10.

^d^ Relative standard deviation, based on five intraday replicate analyses of pure water spiked at two different concentrations.

^e^ Relative standard deviation, based on five interday replicate analyses of pure water spiked at 400 ng L^−1^.

^f^ Based on triplicate analyses of the raw wastewater sample spiked at 400 ng L^−1^.

^g^ Relative standard deviation, based on intraday triplicate analyses of the raw wastewater sample spiked at 400 ng L^−1^.

^h^ Expanded uncertainty (top‐down approach) based on Eurachem/CITAC Guide (2012), with an expansion factor of 2 at a 95% confidence level.

**FIGURE 3:**
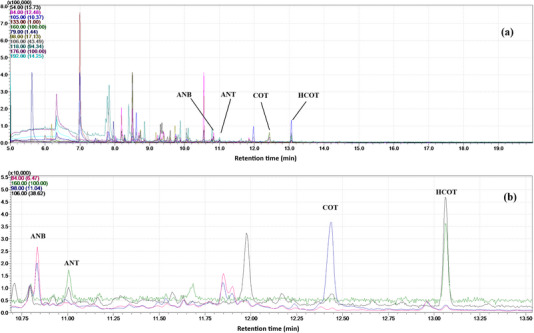
Chromatogram of the raw wastewater sample (a), and mass chromatograms of a pure water sample spiked at 30 ng L^−1^ of ANB and ANT, 90 ng L^−1^ of COT, and 120 ng L^−1^ of HCOT (b).

The raw municipal wastewater sample was spiked at 400 ng L^−1^ and analyzed to evaluate the accuracy of the method in terms of relative recovery. The obtained values (87%–103%) showed that, compared with the pure water sample, the method can still provide reasonably accurate results and overcome the interferences arising from the highly complex matrix of the sample. The uncertainty of the optimized method for spiked real samples was evaluated using a top‐down approach, based on triplicate analyses and an expansion factor of 2 at a 95% confidence level [35]. The resulting uncertainty values ranged from 19.4% for ANB to 31.4% for HCOT, as detailed in Table 2. To provide a brief comparison, a recently published Orbitrap LC–MS approach [16] has achieved LODs of 10–40 ng L^−1^, RSD values of 16.0%–47.9% (at 300 ng L^−1^), and relative recoveries of 48.6%–110.4%, for the same analytes using 50 mL samples. Figure 3 shows a chromatogram of the real sample (a) and mass chromatograms of the spiked pure water (b).

### 3.4 Greenness Assessment

**FIGURE 4:**
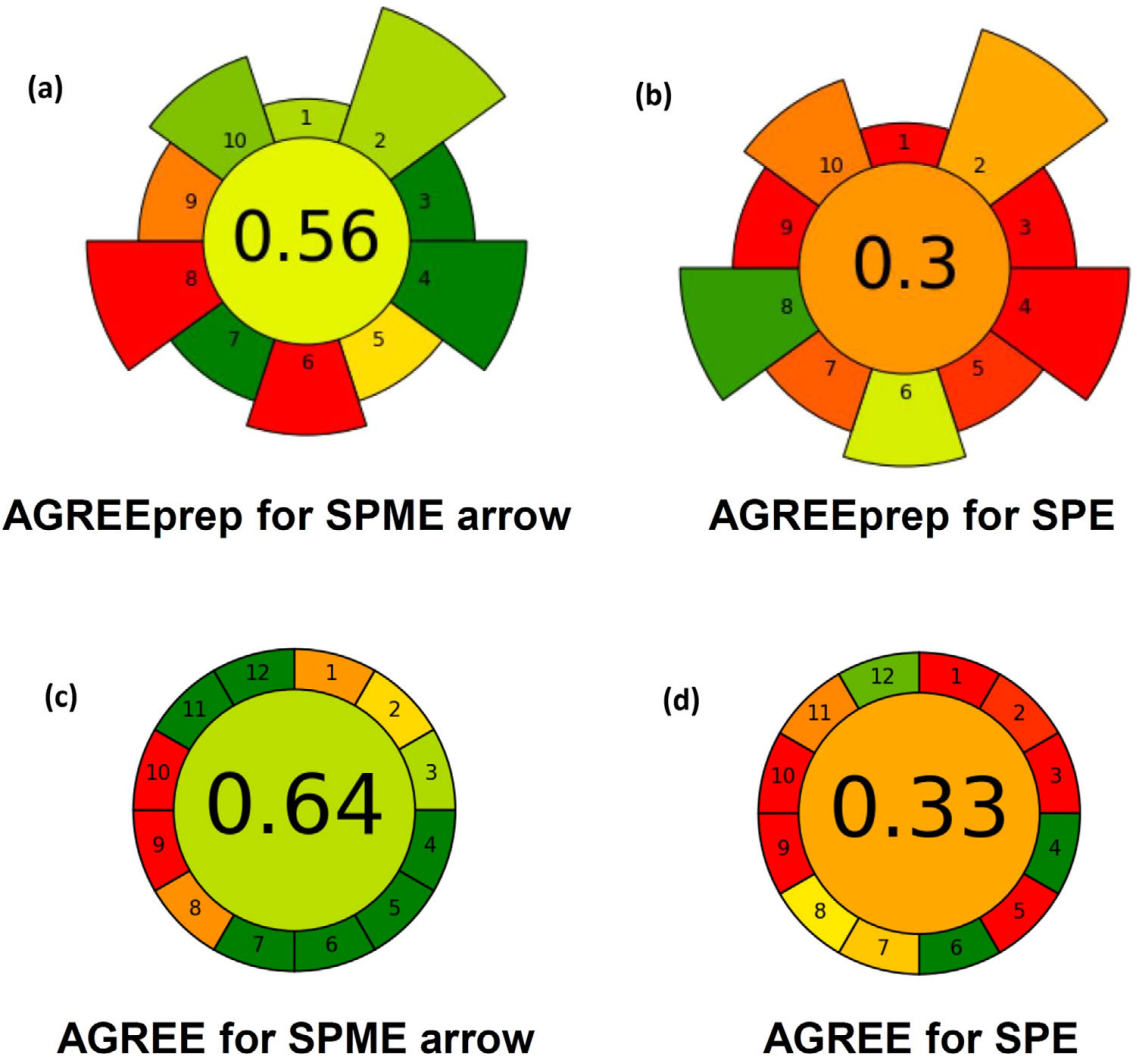
Visual outcomes of AGREE and AGREEprep greenness assessment.

Figure 4 demonstrates the outcomes of the greenness assessment of the developed extraction technique (using the AGREEprep model) as well as the entire analytical procedure (according to the AGREE model). These have also been compared with a method based on SPE followed by an Orbitrap LC–MS analysis [16]. The detailed reports of these assessments are presented in Figures S1–S4, which represent the score of each method for each criterion. Considering Figure 4a,b, the greener performance of SPME Arrow (AGREEprep score of 0.56) over SPE (0.3) is clear, as it is expected from a miniaturized solvent‐free microextraction. However, SPE showed to be superior in two greenness criteria: sample throughput and energy consumption (Criteria 6 and 8, respectively). This is because even in a manual mode of operation, multiple samples could be extracted simultaneously, which is not a convenient approach in SPME. As a result, SPE provides a better sample throughput compared with SPME. Furthermore, the elevated temperature of the samples (here, 65°C) during thermal equilibration and extraction is the reason for the lower score of SPME in energy consumption (0.0), while SPE only requires a laboratory vacuum pump for multiple samples and scores much higher (0.9). It is worth noting that implementing a direct immersion mode for SPME could improve its greenness score by reducing the required energy; however, this would result in losing all the benefits of the headspace mode, as mentioned in Section 3.1.

Figure 4c,d shows that the HS‐SPME Arrow GC–MS method is greener than SPE‐LC–MS, in terms of both individual criteria and the total AGREE score. Automated online SPE‐LC–MS could have a higher score regarding Criterion 5 of the AGREE model (automated and miniaturized methods), but the negative aspect arising from the lack of miniaturization will still remain unresolved.

## 4 Conclusion

The developed technique provided a fully automated procedure capable of sensitive analysis of four crucial nicotine biomarkers in raw municipal wastewater. The headspace mode of extraction helped overcome the interferences caused by the highly complex matrix of the sample, while also allowing the extraction parameters, such as sample pH and ionic strength, as well as stirring rate, to be set without impacting the extraction phase. The sensitivity, precision, linear range, and relative recovery values were comparable with those of previous methods. The optimized method is capable of analyzing approximately 24 samples per day. Considering that raw wastewater samples exhibit high microbial activity, the achieved analytical throughput minimizes the storage time of the collected samples. It also minimizes the sample handling and provides better operator safety conditions. The greenness of the extraction technique, as well as the entire analytical procedure, was assessed using AGREEprep and AGREE models, respectively, and compared with SPE‐Orbitrap LC–MS. The results demonstrated that, except for sample throughput and energy consumption (which is necessary to increase sample temperature), the HS‐SPME Arrow was a greener procedure overall and in all other individual criteria, besides yielding a better AGREE score for the entire analytical procedure. Although the number of individual analytes analyzed in the WBE studies could be higher using LC‐based procedures, the current method can still be considered an automated, sensitive, and environmentally friendly alternative for the studies focused on nicotine biomarkers.

## Author Contribution

**Amir Salemi**: Conceptualization, Formal Analysis, Methodology, Writing Original Draft Preparation. **Merve Çakmakci**: Investigation. **Maryam Vosough**: Software, Writing Review & Editing. **Torsten C. Schmidt**: Supervision, Writing Review & Editing.

## Conflicts of Interest

The authors declare no conflicts of interest.

## Supporting information




**Supporting file 1**: jssc70337‐sup‐0001‐SuppMat.docx

## Data Availability

Research data are not shared.
