# Supplementary material for: Developing a Headspace SPME Arrow GC–MS Method for the Determination of Nicotine Biomarkers in Raw Sewage
Source: J Sep Sci. 2025 Dec 18;48(12):e70337. doi: 10.1002/jssc.70337 (PMC12712886; doi:10.1002/jssc.70337)
Supplement: Supplementary file 1 — Supporting file 1: jssc70337‐sup‐0001‐SuppMat.docx [file JSSC-48-e70337-s001.docx]

*Supporting material for the manuscript submitted to the Journal of Separation Science*

Developing a Headspace SPME Arrow GC-MS Method for the Determination of Nicotine Biomarkers in Raw Sewage
Amir Salemi^a, *^, Merve Çakmakci^b^, Maryam Vosough^a^, Torsten C. Schmidt^a, c^

^a^ Instrumental Analytical Chemistry and Centre for Water and Environmental Research, Faculty of Chemistry, University of Duisburg-Essen, Universitätsstraße 5, 45141 Essen, Germany

^b^ Department of Chemistry, Middle East Technical University, Çankaya 06800, Ankara, Turkey

^c^ IWW Water Centre, Moritzstrasse 26, 45476 Mülheim an der Ruhr, Germany

Corresponding author: Amir Salemi, E-Mail: amir.salemi@uni-due.de


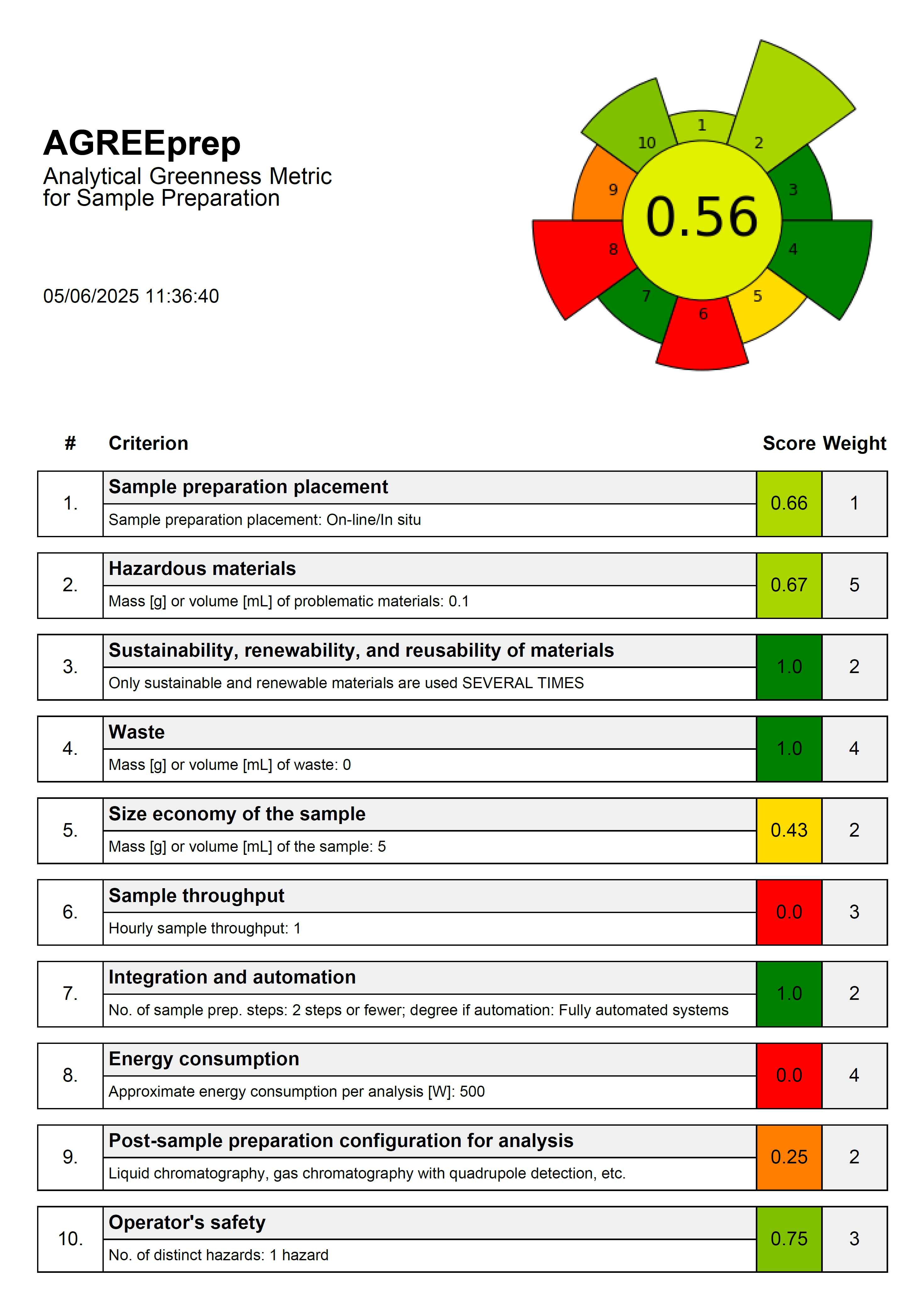


Figure S1. The assessment report of AGREEprep for SPME Arrow


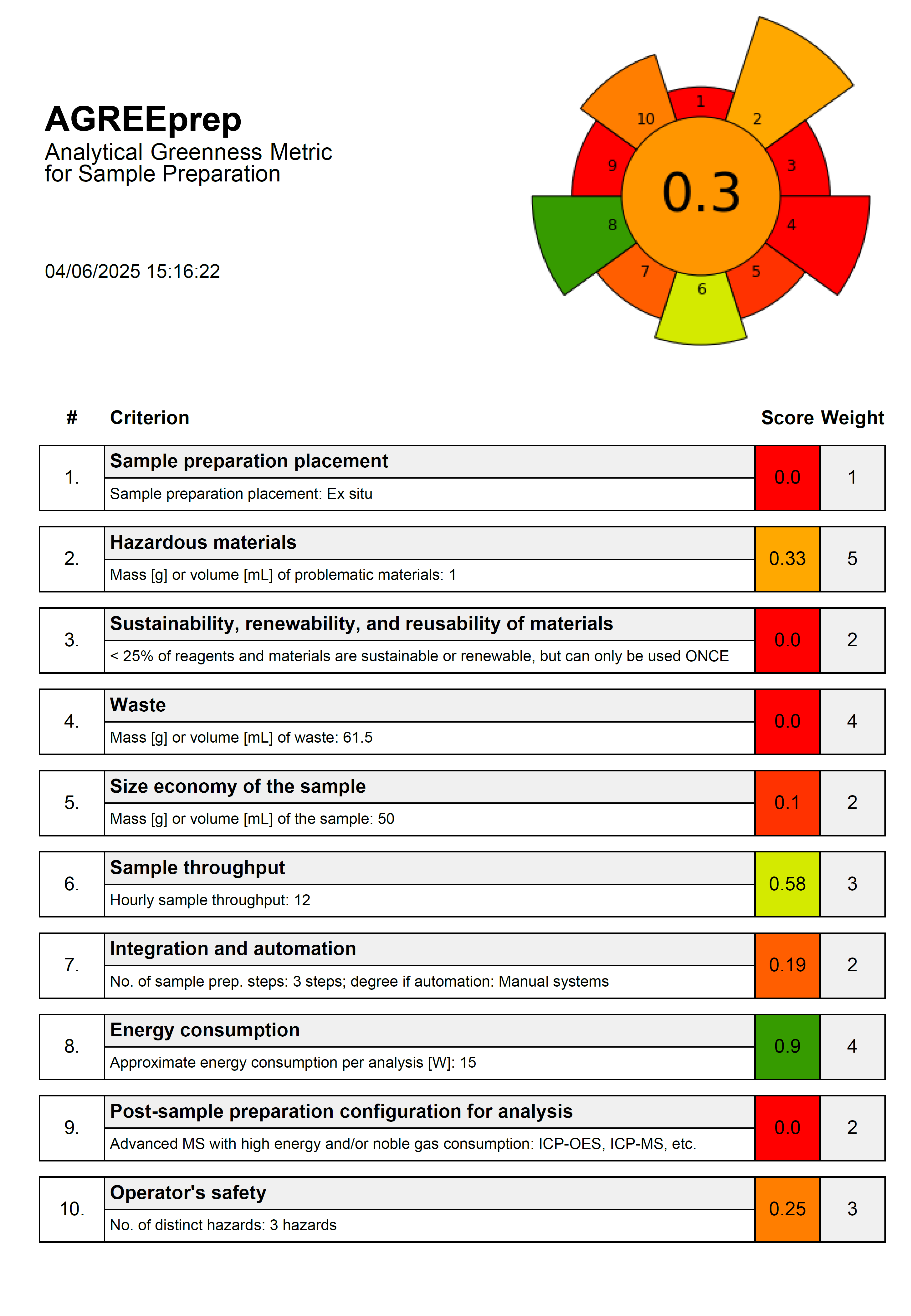


Figure S2. The assessment report of AGREEprep for SPE

Figure S3. The assessment report of AGREE for SPME Arrow

Figure S4. The assessment report of AGREE for SPE
